# Breastfeeding patterns in cohort infants at a high-risk fetal, neonatal and child referral center in Brazil: a correspondence analysis

**DOI:** 10.1186/s12887-020-02272-w

**Published:** 2020-08-07

**Authors:** Maíra Domingues Bernardes Silva, Raquel de Vasconcellos Carvalhaes de Oliveira, José Ueleres Braga, João Aprígio Guerra de Almeida, Enirtes Caetano Prates Melo

**Affiliations:** 1 Human Milk Bank in the Fernandes Figueira Nacional Institute for Women, Children and Adolescent Health (IFF), Oswaldo Cruz Foundation (FIOCRUZ), Av Rui Barbosa, 716, Flamengo, Rio de Janeiro, RJ CEP: 22250-020 Brazil; 2 National Institute of Infectology (FIOCRUZ), Rio de Janeiro, Brazil; 3 National School of Public Health (FIOCRUZ), Rio de Janeiro, Brazil; 4 Global Network of Human Milk Banks (FIOCRUZ), Rio de Janeiro, Brazil

**Keywords:** Longitudinal cohort, Cohort profile, Correspondence analysis, Breastfeeding, High risk

## Abstract

**Background:**

To investigate the prevalence and patterns of breastfeeding at discharge and in the first six months of life in a high-risk fetal, neonatal and child referral center.

**Methods:**

Prospective, longitudinal study that included the following three steps: hospital admission, first visit after hospital discharge and monthly telephone interview until the sixth month of life. The total number of losses was 75 mothers (7.5%). Exposure variables were sorted into four groups: factors related to the newborn, the mother, the health service and breastfeeding. The dependent variable is breastfeeding as per categories established by the WHO. All 1200 children born or transferred to the high-risk fetal, neonatal and child referral center, within a seven-day postpartum period, from March 2017 to April 2018, were considered eligible for the study, and only 1003 were included. The follow-up period ended in October 2018. For this paper, we performed an exploratory analysis at hospital discharge in three stages, as follows: (i) frequencies of baseline characteristics, stratified by risk for newborn; (ii) a multiple correspondence analysis (MCA); and (iii) clusters for variables related to hospital practice and exclusive breastfeeding (EBF).

**Results:**

The prevalence of EBF at hospital discharge was 65.2% (62.1–68.2) and 20.6% (16.5–25.0) in the six months of life. Out of all at-risk newborns, 45.7% were in EBF at discharge. The total inertia corresponding to the two dimensions in the MCA explained for 75.4% of the total data variability, with the identification of four groups, confirmed by the cluster analysis.

**Discussion:**

Our results suggest that robust breastfeeding hospital policies and practices influence the establishment and maintenance of breastfeeding in both healthy and at-risk infants. It is advisable to plan and implement additional strategies to ensure that vulnerable and healthy newborns receive optimal feeding. It is necessary to devote extra effort particularly to at-risk infants who are more vulnerable to negative outcomes.

**Conclusion:**

At-risk newborns did not exclusively breastfeed to the same extent as healthy newborns at hospital discharge. A different approach is required for at-risk neonates, who are more physically challenged and more vulnerable to problems associated with initiation and maintenance of breastfeeding.

## Introduction

The several benefits of breastfeeding for women’s and children’s health as well as short- and long-term economic and environmental benefits to the nation [1] are recognized, and cover populations living in high-, middle- and low-income countries [2]. They apply to both healthy and high-risk children [3, 4]. Despite the available evidence, overall breastfeeding rates remain well below international goals, of at least 50% by 2025 [1, 5, 6].

Globally, breastfeeding rates remain lower than the required to protect the health of women and children. Only 41% of infants under six months of age are exclusively breastfed, and this practice is prevalent (higher than 50%) in only 43 out of 194 countries, always in low or middle-income countries [7]. In Brazil, with approximately 210 million inhabitants and about 2.9 million births per year [8, 9], the last breastfeeding survey, conducted 10 years ago, found a 41% prevalence of exclusive breastfeeding (EBF) among infants under six months of life [10]. Since then, no other research with this scope has been conducted.

Few longitudinal evaluations were identified in a recent systematic review of Brazilian publications on breastfeeding-associated factors [11]. Out of the seven cohorts, five followed children up to the sixth month, and from these, only one cohort had a population higher than 1000 children [12] at baseline; in four cohorts, newborns who were twins, with congenital malformations, low birth weight or hospitalized in a Neonatal Intensive Care Unit (NICU) were excluded. Out of the identified cohorts, none was geared to high-risk hospitals.

The term ‘at-risk newborns’ refers to those exposed to situations with a greater risk of unfavorable development, as they demand special and priority attention [13].

Despite the generally and specifically recognized benefits of breastfeeding and the use of human milk for at-risk infants [14–18], preterm newborns [19] with low birth weight [5], syndrome or with congenital malformations [20] are often not breastfed to the same extent as healthy infants. This subgroup is usually excluded in other published studies, and few longitudinal studies seek to identify and analyze the determinants that influence breastfeeding patterns in at-risk infants.

Consistent evidence indicates that breastfeeding practices are affected by several historical, socioeconomic, cultural and individual factors [5]. In health systems and services, health professionals at all levels influence and support the establishment and maintenance of exclusive and continuous breastfeeding [5]. In a hospital environment, the “Brazilian Human Milk Bank Network”, the “Baby-Friendly Hospital Initiative” (BFHI) and the “Kangaroo Method” components combine and enhance actions to foster the Brazilian policy of promoting, protecting and supporting breastfeeding at this level of care [21]. Previous studies indicated that high-risk infants admitted to the ICU are more likely to benefit from hospital breastfeeding policies implemented through these hospital strategies [22–25].

The Human Milk Bank (HMB) Network provides human milk safely for at-risk newborns, providing clinical assistance in breastfeeding [21]. The Baby-Friendly Hospital Initiative is based on adherence to the Ten Steps to Successful Breastfeeding and has a positive impact on short-, medium- and long-term breastfeeding outcomes [26], and the Kangaroo Method stimulates BF in low birth weight newborns in the maternity ward and in the follow-up after hospital discharge [21].

By knowing the prevalence of breastfeeding for at-risk infants and the relationship of variables related to hospital practice and breastfeeding at discharge, it will be possible to design strategies and actions to improve this outcome.

This study aims to investigate the prevalence and patterns of breastfeeding at discharge and in the first six months of life in a high-risk fetal, neonatal and child referral center.

## Methods/design

A prospective cohort study on breastfeeding practices was carried out with all children born or transferred to the Fernandes Figueira National Institute for Women, Children and Adolescent Health (IFF), Oswaldo Cruz Foundation (FIOCRUZ), within seven days of delivery, from March 2017 to April 2018. The follow-up period ended in October 2018.

The IFF/FIOCRUZ receives newborns and children from all over Brazil, since it is a referral institution for high-risk cases that aims to provide care, education, and research. The IFF/FIOCRUZ, which has been accredited as a Baby-Friendly Hospital since 1999, is equipped with 40 beds for low-complexity neonatal care, and intermediate, intensive and surgical care; it hosts around 1000 deliveries yearly. The IFF/FIOCRUZ is equipped with a Human Milk Bank, and it is a National Referral Center for the Brazilian Network of Human Milk Banks and a Global Referral Center for 23 cooperating countries.

The study collected follow-up data specifically for this cohort rather than just from routine data sources. Out of the 1200 eligible ones, 197 newborns (16.4%) were excluded for the following reasons: (i) mothers had contra-indications for breastfeeding due to conditions of human immunodeficiency virus (HIV) and human T-cell lymphotropic virus (HTLV); (ii) newborns had anencephaly; (iii) newborns had congenital pathology incompatible with life, regarding which the medical team pointed out that it was impossible to provide an oral diet at any stage of life; (iv) indication of gastrostomy in the first week of life; (v) foreign-language speaking mothers, i.e., those who did not understand Portuguese (vi); failing to meet the research assistant, (vii); neonatal death within the first five days of life; (viii) nursing mothers who refused to participate in the study.

The data collection team invited newborns and their volunteer mothers within three days of the birth of the newborn. Out of the 1200 infants born or transferred to our referral center, 154 participants were excluded due to non-eligibility, 30 failed to meet the research assistant and other 13 participants declined the request to take part in the study. The final number of participants included in the study was 1003. The mothers who took part in the study completed the written informed consent and responded to a preliminary interview at the hospital. For participating mothers under the age 18, a parent or guardian provided consent on their behalf. The total number of patients who lost to follow-up within the six months of the original study was 75 mothers (7.5%).

This is a three-phase study. The first phase was performed in the maternity ward through individual interviews, and data was extracted from hospital records during the period of hospitalization after birth, regardless of the length of hospital stay, with collection of hospitalization data (with feeding records in this period), also to obtain sociodemographic characteristics and data related to prenatal care, delivery, women, children and breastfeeding.

In the second phase, mothers were interviewed at the first visit after hospital discharge at the HMB or neonatology follow-up clinic or neurosurgery outpatient clinic (that occurs within 10–15 days of discharge). Telephone interviews were conducted monthly in the last phase until the sixth month of the child’s life. Up to ten telephone contact attempts were made each month to minimize follow-up losses.

A control and quality assurance process was established in data collection and application of research instrument in order to ensure the quality desired for the study results. It was based on data from the literature, as well as professional expertise; training and certification of the data collection team (one pediatrician, two neonatologists, two nursing residents and six nursing students); pretesting of the instruments; a pilot study during the first month; collection with data entry directly in the web application developed for this research accessed on a mobile device or computer with internet access (validation and data analysis, with generation of automatic tabulation, errors and missing reports).

The exposure variables from the hospitalization to the sixth months of life of the child were sorted out into four groups, which is more detailed in an additional file (see Additional file 1 in the online appendix).

For this paper, we performed a three-stage exploratory analysis on hospital discharge. The first included frequencies of baseline characteristics and stratified by risk of newborn. Out of the categories of at-risk newborns of the American Academy of Pediatrics [13], we selected five categories for this study, namely: preterm, low birth weight, surgical anomalies, genetic syndrome or those who required clinical support in the NICU. In this study, potential risk was defined as the existence of at least one gestational morbidity [27]. The definition of potential risk entails the possibility of having a health problem, without necessarily indicating the disease and its probability of occurrence [28].

In the second stage, the joint relationships between factors related to hospital practice and outcome were explored. The variables related to exclusive breastfeeding practices and to hospital practices were selected, and they were defined as: (i) skin-to-skin contact in the first hour of life; (ii) guidance on breastfeeding during prenatal care; (iii) use of a pacifier; and (iv) rooming in (mother and infant remain together 24 h per day). These practices correspond to four steps of the BFHI [29].

The multiple correspondence analysis (MCA) was used to explore joint relationships. MCA is a descriptive dimensionality reduction technique that employs categorical variables. The method used to calculate the inertia was the Burt matrix [30]. The explanatory power of the variability provided ranges from 0 to 100% and the greater the variability, the greater the explanatory power. The number of dimensions generated in the MCA was chosen by the elbow of the graph observed in the scree plot of inertias.

The positions of the categories of each variable in the multidimensional plane can be used to determine groups with similar patterns through graphical representation. Two supplementary variables related to the child and the mother were used for a better understanding of the observed groups: maternal education and risk of the newborn. Then, a hierarchical cluster analysis was performed from the coordinates obtained in the MCA to confirm the verified groups by proximity in the visual inspection.

The R Foundation for Statistical Computing version 3.5.2 was used to analyze data. The *ca* library [30] was employed for this technique, and the *ggplot2* library [31] was used to obtain the MCA graph. The *factoextra* library [32] was used for the dendrogram. This study has been approved by the Ethics Committees at IFF/FIOCRUZ, Brazil (Protocol Number: 1.930.996–2017).

## Results

**Fig. 1:**
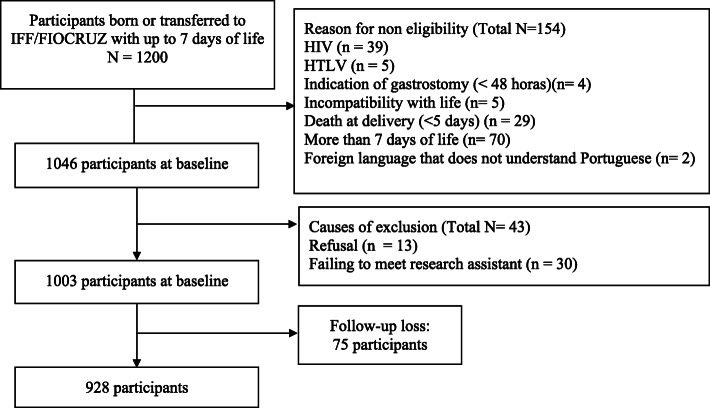
Flowchart of participant selection. FIOCRUZ: Oswaldo Cruz Foundation. HIV: Human Immunodeficiency Virus. HTLV: Human T-cell Lymphotropic Virus. IFF: Fernandes Figueira National Institute for Women, Children and Adolescent Health

A total of 1003 participants was selected for this study. Figure 1 illustrates the flowchart of the selection process of participants for this study. Concerning maternal factors, mothers had a mean age of 27.4 (SD = 6.97); nearly all mothers expressed that they had intended to exclusively breastfeed. Almost half of the households earned less than 2 minimum wages and most women had complete secondary school or incomplete higher education. The main characteristics of the participants in the study were classified per clusters: factors related to mother, child, health service and breastfeeding, as shown in Table 1.

**Table 1:** Baseline characteristics of the 1003 child participants, Rio de Janeiro, Brazil, 2018

Characteristics	n	%	95% CI
**Child-related factors**			
**Sex**			
Female	483	48.2	45.0–51.3
Male	520	51.8	48.6–54.9
**Multiple gestation**			
No	854	85.1	82.7–87.2
Yes	149	14.9	12.7–17.2
**Gestational age**			
Higher or equal to 37 weeks	777	77.5	74.7–80.0
Less than 37 weeks	226	22.5	19.9–25.2
**Birth weight**			
Higher than 2500 g	806	80.4	77.7–82.7
Between 1500 g and 2500 g	158	15.8	13.5–18.1
Lower than 1500 g	39	3.9	2.7–5.2
**Surgical morbidity**^a^			
No	873	87.0	84.8–89.0
Yes	130	13.0	10.9–15.1
**Perinatal morbidity**^b^			
No	589	58.7	55.6–61.7
Yes	414	41.3	38.2–44.3
**Genetic syndrome**			
No	992	98.9	98.0–99.4
Yes	11	1.1	0.5–1.9
**Mother-related factors**			
**Maternal schooling**			
Illiterate / Incomplete elementary school	112	11.2	9.2–13.2
Complete elementary school / Incomplete secondary school	271	27.1	24.2–29.8
Complete secondary School / Incomplete higher education	523	52.3	48.9–55.2
Complete higher education	94	9.4	7.6–11.3
**Parity**			
**Primiparous**	504	50.6	47.1–53.3
**Multiparous**	493	49.4	46.0–52.2
**Number of prenatal care visits**			
> 6 visits	893	89.4	86.9–90.9
< 6 visits	106	10.6	8.7–12.6
**Tobacco use during pregnancy**			
No	914	91.8	89.1–92.9
Yes	82	8.2	6.5–10.0
**Morbidity during pregnancy**			
No	518	51.6	48.4–54.7
Yes	485	48.4	45.2–51.5
**Household income**			
More than 2 MWs	498	60.5	46.5–52.7
Less than 2 MWs	325	39.5	29.5–35.4
**Mothers working outside the home**			
No	545	54.8	51.1–57.4
Yes	449	45.2	41.6–47.9
**Mothers that study**			
No	879	88.1	85.4–89.6
Yes	119	11.9	9.9–14.0
**Maternity leave**^c^			
Yes	445	44.6	41.2–47.5
No	553	55.4	51.9–58.2
**Return to work**			
Six months or more	56	5.6	4.2--7.1
Between four and five months	267	26.7	23.9–29.4
Less than four months	93	9.3	7.5–11.2
Unemployed	512	51.2	47.9–54.1
Did not answer	72	7.2	5.6–8.9
**Living with a partner**			
No	174	17.4	15.0–19.8
Yes	825	82.6	79.7–84.5
**Breastfeeding desire after birth**			
Extreme desire to breastfeed	932	93.0	91.1–94.4
Sometimes prefers a bottle with formula	22	2.2	1.3–3.3
Breastfeeding desire varies	38	3.8	2.6–5.1
Always believes that a bottle with formula is better	10	1.0	0.4–1.8
**Health service-related factors**			
**Skin-to-skin contact in the delivery room**			
Yes	464	46.5	43.1–49.4
No	533	53.5	49.9–56.2
**Place of hospitalization of newborn**			
Rooming-in	674	67.3	64.1–70.1
Neosurgical Intensive Care Unit	55	5.5	4.1–7.0
Neonatal Intensive Care Unit	273	27.2	24.4–30.0
**Received orientation on breastfeeding in prenatal care**			
Yes	718	71.8	68.6–74.3
No	282	28.2	25.3–31.0
**Delivery type**			
Transpelvian	415	41.4	38.3–44.4
Cesarean	588	58.6	55.5–61.9
**Breastfeeding**			
**Feeding at hospital discharge**			
Exclusive breastfeeding	639	65.2	62.1–68.2
Supplemented breastfeeding	272	27.5	24.3–29.9
Bottle feeding	71	7.3	5.5–8.8
**NB received Pasteurized Donated Human Milk**			
No	444	44.3	41.1–47.4
Yes	559	55.7	52.5–58.8
**NB cup fed during hospitalization**			
No	389	38.8	35.7–41.8
Yes	614	61.2	58.1–64.2
**NB bottle fed during hospitalization**			
No	848	84.5	82.1–86.7
Yes	155	15.5	13.2–17.8
**NB used a pacifier during hospitalization**			
No	856	85.7	83.0–87.4
Yes	143	14.3	12.1–16.5

*NB* newborn, *MW* minimum wage (Brazilian monthly minimum wage)

^a^ defined by at least one perinatal morbidity

^b^ defined by at least one surgical anomaly

^c^ the absence of maternity leave included diverse social conditions: no maternity leave and unemployed

Loss of follow-up by patients were caused by non-attendance at hospital discharge, in addition to failure to provide a telephone interview due to incorrect telephone numbers, participants not answering the phone or the phone being out of the coverage area, and busy telephone lines. Concerning patients who lost to follow-up, we did not identify any difference between the participants who were lost and those who remained in the study (see Additional file 2 in the online appendix).

Among participants, 407 (40.6%) newborns were at risk, namely, 226 (22.5) preterm, 197 (19.6%) with low birth weight, 11 (1.1%) with genetic syndrome, and 328 (32.7%) required clinical support from the NICU. Among newborns who were born healthy, almost half (48.4%) had a potential risk at birth due to the presence of at least one gestational morbidity (Table 1). The main gestational morbidities found were urinary tract infection, gestational diabetes, hypertension, pre-eclampsia, syphilis, toxoplasmosis, cytomegalovirus, placenta praevia, and placental abruption.

The prevalence of exclusive breastfeeding at hospital discharge was 65.2% (62.1–68.2) and 20.6% (16.5–25.0) in the six months of life. Out of all newborns at risk, slightly less than half were in EBF at hospital discharge. Table 2 shows the variables related to the mother, the child, the feeding practice and the use of artificial nipples stratified by risk.

**Table 2:** Characteristics of the infant participants stratified by risk classification, Rio de Janeiro, Brazil, 2018

Characteristics	Total ***n*** = 1003			At-Risk Newborn^a^ ***n*** = 407			Healthy Newborn ***n*** = 596			***p***-value
	n	%	95% CI	n	%	95% CI	n	%	95% CI	
**Sex**	1003									0.006
Female		48.2	(45.0–51.3)	174	42.8	(37.9–47.7)	309	51.8	(47.7–55.9)	
Male		51.8	(48.7–55.0)	233	57.2	(52.3–62.1)	287	48.2	(44.1–52.3)	
**Multiple gestation**	1003									< 0.001
No		85.1	(82.8–87.3)	297	73.0	(68.4–77.2)	557	93.5	(91.2–95.3)	
Yes		14.9	(12.7–17.2)	110	27.0	(22.8–31.6)	39	6.5	(4.7–8.8)	
**Perinatal morbidity**	1003									< 0.001
No		58.4	(55.3–61.5)	28	6.9	(4.6–9.8)	558	93.6	(91.4–95.4)	
Yes		41.6	(38.5–44.7)	379	93.1	(90.2–95.4)	38	6.4	(4.6–8.6)	
**Maternal age**	1000									0.98
20–34 years		68.7	(65.7–71.6)	279	68.6	(63.8–73.0)	408	68.8	(64.9–72.5)	
< 20 years		13.9	(11.8–16.2)	56	13.8	(10.6–17.5)	83	14.0	(11.3–17.1)	
> 35 years		17.4	(15.1–19.9)	72	17.7	(14.1–21.8)	102	17.2	(14.2–20.5)	
**Maternal schooling**	1000									0.06
Illiterate		11.2	(9.3–13.3)	51	12.5	(9.5–16.1)	61	10.3	(8.0–13.0)	
Elementary school		27.1	(24.4–30.0)	112	27.5	(23.2–32.1)	159	26.8	(23.3–30.6)	
Secondary school		52.3	(49.2–55.4)	196	48.2	(43.2–53.1)	327	55.1	(51.0–59.2)	
Higher education		9.4	(7.7–11.4)	48	11.8	(8.8–15.3)	46	7.8	(5.7–10.2)	
**Received orientation on BF in prenatal care**	1000									0.001
Yes		71.8	(68.9–74.6)	269	66.1	(61.3–70.7)	449	75.7	(72.1–79.1)	
No		28.2	(25.4–31.1)	138	33.9	(29.3–38.7)	144	24.3	(20.9–27.9)	
**Rooming-in**	999									< 0.001
Yes		70.8	(67.8–73.6)	137	33.8	(29.2–38.7)	570	96.0	(94.0–97.4)	
No		29.2	(26.4–32.2)	268	66.2	(61.3–70.8)	24	4.0	(2.6–6.0)	
**Skin-to-skin contact in the delivery room**	997									< 0.001
Yes		46.5	(43.4–49.7)	97	23.9	(19.8–28.3)	367	62.1	(58.0–66.0)	
No		53.5	(50.3–56.6)	309	76.1	(71.7–80.2)	224	37.9	(34.0–42.0)	
**Morbidity during pregnancy**	1003									0.002
No		51.6	(48.5–54.8)	186	45.7	(40.8–50.7)	332	55.7	(51.6–59.7)	
Yes		48.4	(45.2–51.5)	221	54.3	(49.3–59.2)	264	44.3	(40.3–48.4)	
**BF desire after birth**	1002									0.63
Strong desire		93.0	(91.3–94.5)	381	93.6	(90.8–95.8)	551	92.6	(90.2–94.6)	
Weak desire		7.0	(5.5–8.7)	26	6.4	(4.2–9.2)	44	7.4	(5.4–9.8)	
**Delivery type**	1003									< 0.001
Cesarean		58.6	(55.5–61.7)	301	74.0	(69.4–78.2)	287	48.2	(44.1–52.3)	
Transpelvian		41.4	(38.3–44.5)	106	26.0	(21.8–30.6)	309	51.8	(47.7–55.9)	
**Place of hospitalization of newborn**	1002									< 0.001
Maternal Care		68.4	(65.4–71.2)	90	22.1	(18.2–26.5)	595	100.	0 (99.4–100.0)	
Neonatal Intensive Care Unit		31.6	(28.8–34.6)	317	77.9	(73.5–81.8)	0	0.0	(0.0–0.6)	
**Difficulty BF**	1003									< 0.001
No		40.0	(36.9–43.1)	113	27.8	(23.5–32.4)	288	48.3	(44.2–52.4)	
Yes		60.0	(56.9–63.1)	294	72.2	(67.6–76.5)	308	51.7	(47.6–55.8)	
**NB used a pacifier during hospitalization**	999									< 0.001
No		85.7	(83.4–87.8)	273	67.6	(62.8–72.1)	583	98.0	(96.5–99.0)	
Yes		14.3	(12.2–16.6)	131	32.4	(27.9–37.2)	12	2.0	(1.0–3.5)	
**NB received Pasteurized Donated Human Milk**	1003									< 0.001
No		44.3	(41.2–47.4)	77	18.9	(15.2–23.1)	367	61.6	(57.5–65.5)	
Yes		55.7	(52.6–58.8)	330	81.1	(76.9–84.8)	229	38.4	(34.5–42.5)	
**NB received formula**	1003									< 0.001
No		64.7	(61.7–67.7)	183	45.0	(40.1–49.9)	466	78.2	(74.7–81.4)	
Yes		35.3	(32.3–38.3)	224	55.0	(50.1–59.9)	130	21.8	(18.6–25.3)	
**NB cup fed during hospitalization**	1003									0.01
No		38.8	(35.8–41.9)	138	33.9	(29.3–38.7)	251	42.1	(38.1–46.2)	
Yes		61.2	(58.1–64.2)	269	66.1	(61.3–70.7)	345	57.9	(53.8–61.9)	
**NB bottle fed during hospitalization**	1003									< 0.001
No		84.5	(82.2–86.7)	254	62.4	(57.5–67.1)	594	99.7	(98.8–100.0)	
Yes		15.5	(13.3–17.8)	153	37.6	(32.9–42.5)	2	0.3	(0.0–1.2)	
**Exposure to a combination of four BFHI steps**	1003									< 0.001
Yes		29.6	(26.8–32.5)	36	8.8	(6.3–12.0)	261	43.8	(39.8–47.9)	
No		70.4	(67.5–73.2)	371	91.2	(88.0–93.7)	335	56.2	(52.1–60.2)	
**Exposure to a combination of two BFHI steps**	1003									< 0.001
Yes		53.3	(50.2–56.5)	104	25.6	(21.4–30.1)	431	72.3	(68.5–75.9)	
No		46.7	(43.5–49.8)	303	74.4	(69.9–78.6)	165	27.7	(24.1–31.5)	
**Feeding practice at hospital discharge**	983									< 0.001
EBF		65.2	(62.1–68.2)	177	45.7	(40.7–50.8)	464	77.9	(74.3–81.1)	
EBF Cessation		34.8	(31.8–37.9)	210	54.3	(49.2–59.3)	132	22.1	(18.9–25.7)	

*CI* Confidence interval

*BF* Breastfeeding

*BFHI* Baby-Friendly Hospital Initiative

*EBF* Exclusive Breastfeeding

*NB* Newborn

^a^At-risk infants: at least one positive characteristic: ‘hospitalization in the neonatal intensive care unit’, ‘prematurity’, ‘low birth weight (< 2500 g)’, ‘Apgar in the fifth minute’, ‘presence of one perinatal morbidity’, ‘presence of one surgical morbidity’ and ‘genetic syndrome

Virtually all newborns were exposed to a minimum of one baby-friendly practice during their hospitalization. Most of the infants did not use a pacifier and approximately half of them immediately initiated skin-to-skin contact in the postpartum period. Most women received guidance on benefits and management of breastfeeding during prenatal care at HMB. In the group where no guidance was provided, over 70% did not have prenatal care at the institute. Two-thirds of the newborns spent 24 h with their mothers (Table 2).

When the four baby-friendly steps were combined, we observed 29.6% (26.8–32.5) of newborns and women who were exposed to four practices. The group of newborns exposed to the four baby-friendly steps had a higher prevalence of EBF at discharge (83.8%) in comparison to the group of newborns with combined exposure of just two steps (rooming in and prenatal breastfeeding information) (76.2%) (Table 2).

The first hospital visit up to 15 days after discharge was carried out with over 50% of the families with the HMB medical and nursing staff, a practice which corresponds to the tenth step of the baby-friendly hospital initiative. Many families did not attend the first visit for financial reasons.

**Fig. 2:**
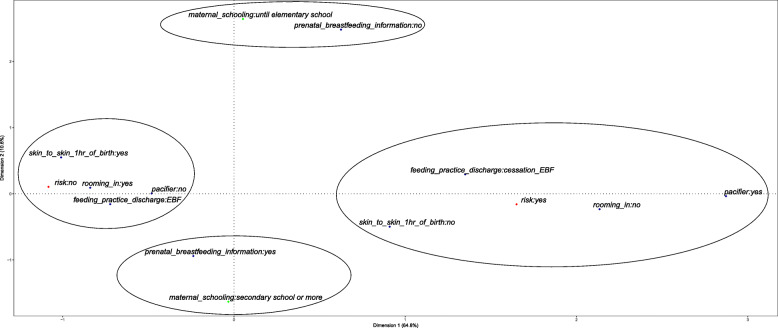
Multiple correspondence analysis of 964 newborns at a high-risk institution, Brazil, 2018. Note: the green color represents the supplementary variable ‘maternal education’ and the red color represents the supplementary variable ‘risk of the newborn’. EBF: Exclusive Breastfeeding

The graphic representation of MCA shows the characteristics related to breastfeeding and hospital practices in two dimensions. The total inertia corresponding to the two dimensions determined by the scree plot explained 75.4% of the total variability of the data. Considering the first dimension, which explains 64.8% of the variability, we observed that components that were favorable to exclusive breastfeeding are positioned in the negative value of dimension 1, while the opposite characteristics related to the cessation of exclusive breastfeeding are located in the positive values of dimension 1. The second dimension explained only 10.6% of the variability (Fig. 2).

**Fig. 3:**
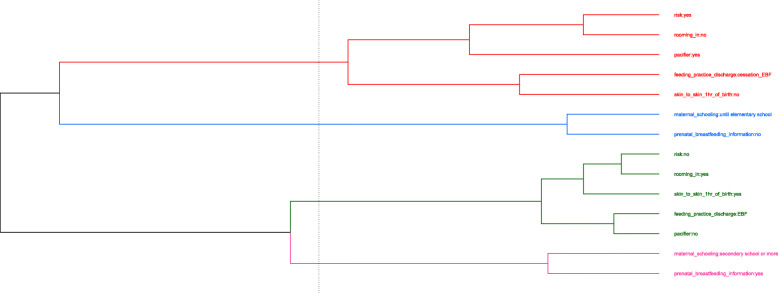
Dendrogram of the cluster analysis of 964 newborns at a high-risk institution, Brazil, 2018. Note: Cluster analysis of the coordinates of the multiple correspondence analysis. EBF: Exclusive Breastfeeding

We observed four groups in Fig. 2 through the joint analysis of the two dimensions: Group A, defined by the characteristics favorable to EBF and proximity to the supplementary variable healthy newborns; Group B, defined by the cessation of EBF, absence of skin-to-skin contact immediately after delivery, use of a pacifier, separation of mother and baby for more than 12 h in the first week positioned close to the supplementary newborn risk variable; Group C was characterized by breastfeeding guidance in prenatal care near the supplementary variable ‘higher maternal education’; and Group D was defined by the group that did not receive guidance on breastfeeding during pregnancy near the supplementary variable ‘low maternal education’. The cluster analysis confirmed the groups found (Fig. 3).

## Discussion

This study showed that hospital practices described four patterns concerning the establishment of EBF at hospital discharge in newborns from a high-risk institution. As expected, favorable hospital practices were associated with exclusive breastfeeding, while the unfavorable ones were grouped with the cessation of this feeding practice at hospital discharge. Against expectations, guidance on the benefits of breastfeeding during prenatal care was not related to the outcome at hospital discharge. Our results suggest that despite the risk or potential risk of the newborn, hospital practices influence the establishment and maintenance of breastfeeding.

Almost half of the studied newborns were considered at risk (no observational study evaluated breastfeeding in great variability in risk exposures in Brazil [11]); and among healthy newborns, almost half had a potential risk at birth due to the presence of gestational morbidity. At hospital discharge, approximately half of the newborns at risk were exclusively breastfed. The cohort of infants was recruited from a referral institution for high fetal, neonatal and child risk. It should be noted that no observational study has evaluated breastfeeding in a wide range of risk exposures in Brazil, a country with a continental dimension marked by contrasts that include income distribution.

At least one baby-friendly practice was applied to virtually all newborns during their hospitalization. A positive dose-response effect concerning the number of baby-friendly practices (in which the mother and the newborn are exposed) and the proportion of newborns exclusively breastfed at hospital discharge was found. This result is similar to the recent systematic review [26] on the impact of BFHI steps on the breastfeeding outcome.

The EBF rate for at-risk newborns observed in this study is higher than the rate shown in four other studies: two Brazilian studies with preterm and low birth weight (5.5 and 39%, respectively) newborns, from which the first study was carried out at IFF/FIOCRUZ [33, 34]; one study held in Japan (22.6%) [35]; and another one in Italy (28%) (28%) [36].

Brazil stands out internationally concerning the development of policies and programs to promote, protect and support breastfeeding [5]. In particular, several efforts have been made over time in the studied institution. The Human Milk Bank distributes pasteurized human milk with certified quality assurance, providing specialized clinical assistance in breastfeeding and monitoring of all hospitalized newborns, besides offering educational groups for pregnant women and families during prenatal care, personalized visits for pregnant women with at-risk newborns, as well as performing the first visit after hospital discharge focused on breastfeeding. Moreover, the institution is committed to maintaining the title “child-friendly hospital”, as accredited in 1999, since it has always been promoting and supporting breastfeeding.

The groups identified in the correspondence analysis showed a pattern similar to other studies in which friendly paediatric breastfeeding practices may demonstrate a positive effect on breastfeeding at hospital discharge [19, 37], an important condition for maintaining this practice [38].

Although few infants were given pacifiers (most were high-risk newborns), ideally, no infants should have access to these accessories, according to the *United Nations Convention on the Rights of the Child (*UNICEF)/*World Health Organization* (WHO) policy [29].

A prospective observational study with 1488 preterm infants revealed that minimizing the use of pacifiers during the transition to the breast, stimulating skin-to-skin contact in stable newborns and rooming-in of the newborn with the mother were associated with the early establishment of breastfeeding and assurance of better rates at hospital discharge in this specific group [19, 39].

Skin-to-skin contact immediately after delivery was not widely used in three thirds of at-risk infants. This result is similar to that found in another cohort of preterm infants [19]. Mothers who are unable to initiate breastfeeding during the first hour after delivery should still be supported to breastfeed as soon as they are able to [40].

Over a half of the newborns attended the specialized visit on breastfeeding at the HMB within 15 days of hospital discharge. This return is intended to assess possible difficulties, aiming to support and provide clinical support in breastfeeding before a team of specialists with expertise in breastfeeding at the HMB. Such hospital routine is essential to encourage the maintenance of exclusive breastfeeding, or transition from complementary to exclusive breastfeeding, with follow-up visits when necessary, as per step 10 of the baby-friendly hospital initiative.

On the other hand, substantial difficulties were found regarding some practices. Concerning the group of healthy newborns, approximately one-third were not exposed to immediate skin-to-skin contact (SSC) with their mothers after delivery. Despite its known benefits, the practice of SSC varies substantially across the world [41]. About one-third of women did not receive guidance on the benefits of breastfeeding (out of these, 73% did not perform prenatal care at IFF/FIOCRUZ), a hospital practice related to prenatal care that was located close to the supplementary variable ‘low maternal education’ in MCA, possibly justifying the low frequency of prenatal care visits, as highlighted by studies carried out in Brazil [42, 43]. Among at-risk newborns, half of them received supplemental feeding at the hospital, as one-fifth of healthy newborns did. Hospital supplementation of breastfeeding infants is associated with delayed onset of lactation, suboptimal breastfeeding practices, perceived problems with breastfeeding during hospital stay, mothers’ perception of insufficient milk supply and shorter exclusive breastfeeding time [44–46]. Over a half of the women were unable to stay 24 h a day with the hospitalized newborn. There is some evidence from similar studies that this practice may have an adverse effect on the establishment and maintenance of EBF [18, 19, 22, 35, 45].

These findings reveal opportunities for improvement and raise a question: why practices that are repeatedly evidenced as relevant to increase breastfeeding rates are not yet established in the same proportion with high-risk groups?

We require actions to support the strengthening of institutional breastfeeding culture, regardless of the risk level of the newborn. Health professionals, in different positions, are aware of the importance of breastfeeding and the use of human milk, but practices and behavior are not always consistent with strengthening the breastfeeding culture. Such culture guides values, attitudes, perceptions, competences and behavior of health care providers with an emphasis on breastfeeding, a practice that favors early discharge, reduces re-hospitalization and morbidities after hospitalization, providing lifelong benefits for these small individuals.

A percentage of 93% women studied reported a desire to breastfeed, and 65% were on EBF at discharge. The best efforts must be made to achieve higher rates, and additional strategies must be planned and implemented in the service and practice of healthcare providers to ensure that the hospital experience can contribute to the promotion of breastfeeding, and that vulnerable and healthy newborns receive optimal feeding.

Strengths of this study include: (i) significant number of participants; (ii) significant time and frequency of follow-up; (iii) a study scenario that provides great variability of the exposures studied in the child risk context: newborn twins/triplets/quadruplets (particularly considering that mother-related factors are equivalent); newborns with congenital malformations, premature or low birth weight, a group in which there is a substantial investment of the institution and its health professionals to strengthen breastfeeding support. In general, such a distribution profile during hospitalization is not concentrated in a single institution, and it is spread at several maternity hospitals; IFF/FIOCRUZ is equipped with resources and technologies necessary for the follow-up of these children; (iv) a control and quality assurance process established in data collection; (v) a qualified data collection team; (vi) hospital record data from an educational, care and research institution; (vi) low dropout rates in research participation (< 5% at 6 months); and (vii) high adherence to research participation (7.5% loss). The limitation may be that results obtained from the analysis with healthy children may not apply to other populations, such as non-specialized maternity hospitals. This is the first prospective study on breastfeeding conducted in Brazil with variability and representativeness of several risk categories. Subsequent publications will be made to assess the effect of determinants on the duration of breastfeeding in this context.

## Conclusion

This study confirms the relationship between hospital practices and the establishment of breastfeeding at hospital discharge. At-risk newborns did not exclusively breastfeed to the same extent as healthy newborns at discharge. Moreover, this group did not experience friendly paediatric breastfeeding practices in the same proportion as healthy newborns, which can be an impediment to the timely initiation of breastfeeding and a failure of this feeding practice at hospital discharge.

A different approach is required for at-risk neonates, who have greater health challenges and are more vulnerable to problems associated with initiation and maintenance of breastfeeding. Health services and providers are co-responsible for the successful practice of mothers who desire to breastfeed. Therefore, hospital practices and services must be revised to ensure the success of EBF at hospital discharge, which is crucial to sustain this feeding practice for a longer time.

The many benefits evidenced continuously must mobilize a joint effort and the encouragement of breastfeeding in each action, conduct and care performed, regardless of the newborn’s risk. Thus, it is necessary to embed a strong breastfeeding promotion, support, and protection culture in high-risk hospitals, reducing the morbidity of at-risk survivors at birth.

## Supplementary information

**Additional file 1.** Summary of variables collected in the breastfeeding cohort, Brazil, 2018.

**Additional file 2.** Comparison between 928 participants and 75 non-respondents. Rio de Janeiro, Brazil, 2018.

## Data Availability

All relevant data are in this paper. The datasets generated and/or analyzed during the current study are not publicly available due The Ethics Committees restricted the full data disclosure since this would compromise participant confidentiality but are available from the corresponding author on reasonable request. We welcome data analysis and publication collaboration through specific research proposals sent to the lead researcher and her co-tutors. Additional information can be obtained by email to maira.silva@iff.fiocruz.br

## Abbreviations

BFHI: Baby-Friendly Hospital Initiative
EBF: Exclusive Breastfeeding
FIOCRUZ: Oswaldo Cruz Foundation
HIV: Human Immunodeficiency Virus
HTLV: Human T-cell Lymphotropic Virus
HMB: Human Milk Bank
IFF: Fernandes Figueira National Institute for Women, Children and Adolescent Health
MCA: Multiple Correspondence Analysis
NICU: Neonatal Intensive Care Unit
SSC: Skin-to-skin contact
UNICEF: United Nations Convention on the Rights of the Child
WHO: World Health Organization

## References

[1] World Health Organization. The investment case for breastfeeding: nurturing the health and wealth of nations [Internet]. UNICEF; 2017 [cited 2018 Jul 21]. Available from: http://www.who.int/nutrition/publications/infantfeeding/global-bf-collective-investmentcase.pdf.

[2] Victora CG, Bahl R, Barros AJD, França GVA, Horton S, Krasevec J (2016). Breastfeeding in the 21st century: epidemiology, mechanisms, and lifelong effect. Lancet..

[3] Sankar MJ, Sinha B, Chowdhury R, Bhandari N, Taneja S, Martines J (2015). Optimal breastfeeding practices and infant and child mortality: a systematic review and meta-analysis. Acta Paediatr.

[4] Alyahya W, Barnett D, Cooper A, et al. Donated human milk use and subsequent feeding pattern in neonatal units. Int Breastfeed J. 2019;14(39). 10.1186/s13006-019-0233-x. 10.1186/s13006-019-0233-x PMC6721171 31507645

[5] Rollins NC, Bhandari N, Hajeebhoy N, Horton S, Lutter CK, Martines JC (2016). Why invest, and what it will take to improve breastfeeding practices?. Lancet.

[6] Global Nutrition Report. Global Nutrition Report: Shining a light to spur action on nutrition. [Internet]. 2018 [cited 2019 Dec 1]. Available from: https://globalnutritionreport.org/reports/global-nutrition-report-2018/.

[7] World Health Organization, UNICEF. Global Breastfeeding Scorecard, 2019 [Internet]. [cited 2020 Jan 4]. Available from: https://www.who.int/nutrition/publications/infantfeeding/global-bf-scorecard-2019/en/.

[8] Ministério de Saúde/SVS/DASIS - Sistema de Informações sobre Nascidos Vivos - SINASC/BRASIL.TabNet Win32 3.0: [Internet]. [cited 2019 Dec 10]. Available from: http://tabnet.datasus.gov.br/cgi/tabcgi.exe?sinasc/cnv/nvuf.def.

[9] Sistema IBGE de Recuperação Automática - SIDRA [Internet]. [cited 2020 Jan 4]. Available from: https://sidra.ibge.gov.br/pesquisa/censo-demografico/demografico-2010/inicial.

[10] Brasil (2009). Ministério da Saúde. Secretaria de Atenção à Saúde. Departamento de Ações Programáticas Estratégicas. II Pesquisa de prevalência de aleitamento materno nas capitais brasileiras e Distrito Federal.

[11] Boccolini CS, de Carvalho ML, de Oliveira MIC (2015). Factors associated with exclusive breastfeeding in the first six months of life in Brazil: a systematic review. Rev. Saúde Pública.

[12] Vieira TO, Silva LR, Vieira GO, de Oliveira NF, Mendes CM, Giugliani ER (2014). Duration of exclusive breastfeeding in a Brazilian population: new determinants in a cohort study. BMC Pregnancy Childbirth.

[13] American Academy of Pediatrics (2008). Hospital discharge of the high-risk neonate. Pediatrics.

[14] Dritsakou K, Liosis G, Valsami G, Polychronopoulos E, Skouroliakou M (2017). The impact of maternal- and neonatal-associated factors on human milk’s macronutrients and energy. J Matern Fetal Neonatal Med.

[15] Scime NV, Burke SM (2018). Environmental Scan of Breastfeeding Resources in Canadian Neonatal Intensive Care Units. J Obstet Gynecol Neonatal Nurs.

[16] Shah PS, Herbozo C, Aliwalas LL, Shah VS (2012). Breastfeeding or breast milk for procedural pain in neonates. Cochrane Database Syst Rev.

[17] Underwood MA (2013). Human milk for the premature infant. Pediatr Clin N Am.

[18] Manzoni P, Stolfi I, Pedicino R, Vagnarelli F, Mosca F, Pugni L (2013). Human milk feeding prevents retinopathy of prematurity (ROP) in preterm VLBW neonates. Early Hum Dev.

[19] Maastrup R, Hansen BM, Kronborg H, Bojesen SN, Hallum K, Frandsen A (2014). Factors associated with exclusive breastfeeding of preterm infants. results from a prospective national cohort study. PLOS ONE.

[20] Salvatori G, Foligno S, Occasi F, Pannone V, Valentini GB, Dall’Oglio I (2014). Human milk and breastfeeding in surgical infants. Breastfeed Med.

[21] Brasil. Ministério da Saúde. Secretaria de Atenção à Saúde. Departamento de Ações Programáticas Estratégicas. Bases para a Discussão da Política Nacional de Promoção, Proteção e Apoio ao Aleitamento Materno. Brasília: Ministério da Saúde, 2017.

[22] Mekonnen AG, Yehualashet SS, Bayleyegn AD. The effects of kangaroo mother care on the time to breastfeeding initiation among preterm and LBW infants: a meta-analysis of published studies. Int Breastfeed J. 2019;14(12). 10.1186/s13006-019-0206-0. 10.1186/s13006-019-0206-0 PMC6379962 30820239

[23] Maastrup R, Bojesen SN, Kronborg H, Hallström I (2012). Breastfeeding support in neonatal intensive care: a national survey. J Hum Lact.

[24] Berti E, Puglia M, Perugi S, Gagliardi L, Bosi C, Ingargiola A (2018). Feeding practices in very preterm and very low birth weight infants in an area where a network of human milk banks is in place. Front Pediatr.

[25] Arslanoglu S, Moro GE, Bellù R, Turoli D, De Nisi G, Tonetto P (2013). Presence of human milk bank is associated with elevated rate of exclusive breastfeeding in VLBW infants. J Perinat Med.

[26] Pérez-Escamila R, Martinez JL, Segura-Pérez S (2016). Impact of the baby-friendly hospital initiative on breastfeeding and child health outcomes: a systematic review. Matern Child Nutr.

[27] Zhu X, Niu H, Wang H, Li X, Qi T, Ding W (2019). High risk pregnancy associated perinatal morbidity and mortality: a second birth population-based survey in Huai’an in 2015. BMC Pregnancy Childbirth.

[28] Costa EA, org. Vigilância Sanitária: temas para debate [online]. Salvador: EDUFBA, 2009. 237 p. ISBN 978–85–232-0652-9. [cited 2019 Dec 2]. Available from SciELO Books: http://books.scielo.org/id/6bmrk.

[29] World Health Organization. Baby-Friendly Hospital Initiative: Revised, Updated and Expanded for Integrated Care [Internet]. Geneva: World Health Organization; 2009 [cited 2019 Dec 2]. Available from: http://www.ncbi.nlm.nih.gov/books/NBK153471/. 23926623

[30] Nenadic O, Greenacre M (2007). Correspondence analysis in R, with two- and three-dimensional graphics: the ca package. J Stat Softw.

[31] Wickham H, Chang W, Henry L, Pedersen TL, Takahashi K, Wilke C, Woo K, Yutani. Elegant Graphics for Data Analysis. The ggplot2 Package, 2019 [Internet]. [cited 2019 Dec 1]. Available from: https://cran.r-project.org/web/packages/ggplot2/ggplot2.pdf.

[32] Kassambara A, Mundt F. Extract and Visualize the Results of Multivariate Data Analyses. The factoextra Package. 2019; [cited 2020 Jan 13]. Available from: https://cran.r-project.org/web/packages/factoextra/factoextra.pdf.

[33] Bicalho-Mancini PG, Velásquez-Meléndez G (2004). Exclusive breastfeeding at the point of discharge of high-risk newborns at a Neonatal Intensive Care Unit and the factors associated with this practice. J Pediatr (Rio J).

[34] Méio MD, Villela LD, Gomes Júnior SCDS, Tovar CM, Moreira MEL. Breastfeeding of preterm newborn infants following hospital discharge: follow-up during the first year of life. Ciência & Saúde Coletiva. 2018; 23(7):2403–12. 10.1590/1413-81232018237.15742016 30020392

[35] Mamemoto K, Kubota M, Nagai A, Takahashi Y, Kamamoto T, Minowa H (2013). Factors associated with exclusive breastfeeding in low birth weight infants at NICU discharge and the start of complementary feeding. Asia Pac J Clin Nutr.

[36] Davanzo R, Monasta L, Ronfani L, Brovedani P, Demarini S (2013). Breastfeeding in neonatal intensive care unit study group. Breastfeeding at NICU discharge: a multicenter Italian study. J Hum Lact.

[37] Alonso-Díaz C, Utrera-Torres I, de Alba-Romero C, Flores-Antón B, Lora-Pablos D, Pallás-Alonso CR (2016). Breastfeeding support in Spanish neonatal intensive care units and the baby-friendly hospital initiative: a national survey. J Hum Lact.

[38] World Health Organization. Implementation guidance: protecting, promoting and supporting breastfeeding in facilities providing maternity and newborn services – the revised Baby-friendly Hospital Initiative. Geneva: World Health Organization; 2018. [cited 2019 Dec 22]. Available from: https://apps.who.int/iris/bitstream/handle/10665/272943/9789241513807-eng.pdf.

[39] Renfrew M, Craig D, Dyson L, McCormick F, Rice S, King S (2009). Breastfeeding promotion for infants in neonatal units: a systematic review and economic analysis. Health Technol Asses.

[40] World Health Organization (2011). Guidelines on optimal feeding of low birth weight infants in low- and middle-income countries.

[41] Abdulghani N, Edvardsson K, Amir LH (2018). Worldwide prevalence of mother-infant skin-to-skin contact after vaginal birth: a systematic review. PLoS One.

[42] O’Connor M, Allen J, Kelly J, Gao Y, Kildea S (2018). Predictors of breastfeeding exclusivity and duration in a hospital without baby friendly hospital initiative accreditation: a prospective cohort study. Women Birth.

[43] Declercq E, Labbok MH, Sakala C, O’Hara M (2009). Hospital practices and women’s likelihood of fulfilling their intention to exclusively breastfeed. Am J Public Health.

[44] Maayan-Metzger A, Avivi S, Schushan-Eisen I, Kuint J (2012). Human milk versus formula feeding among preterm infants: short-term outcomes. Am J Perinatol.

[45] McCoy MB, Heggie P (2020). In-hospital formula feeding and breastfeeding duration. Pediatrics.

[46] Feldman-winter L, Kellams A (2020). In hospital formula feeding and breastfeeding duration. Pediatrics.

