# Supplementary material for: Breastfeeding patterns in cohort infants at a high-risk fetal, neonatal and child referral center in Brazil: a correspondence analysis
Source: BMC Pediatr. 2020 Aug 7;20:372. doi: 10.1186/s12887-020-02272-w (PMC7412808; doi:10.1186/s12887-020-02272-w)
Supplement: Supplementary file 1 — Additional file 1. Summary of variables collected in the breastfeeding cohort, Brazil, 2018. [file 12887_2020_2272_MOESM1_ESM.docx]

**Additional file 1. Summary of variables collected in the breastfeeding cohort, Brazil, 2018.**

| **Questionnaire** | **Variables** | **Data source (time of data collection)** |
| --- | --- | --- |
| Mother-related factors | | |
| Demographic/Social | age, marital status, income, education, employment, maternity leave, return to work | I |
| Lifestyle | Tobacco use during the antenatal period and after birth | I |
| Obstetric | Parity, morbidity during pregnancy, number of prenatal care visits, prenatal care visit place | I |
| Health service-related factors | | |
| During hospitalization | Delivery type, breastfeeding in the first hour of life, skin-to-skin contact in the first hour of life and during Intensive Care Unit admission, place of hospitalization of child (Rooming-in or Neonatal Intensive Care Unit (NICU), mother-baby separation for more than 12 hours in the first hour of life, hospital stay time, maintaining lactation during hospitalization of the baby at fasting, frequency of massage and milk extraction during hospitalization of the baby at fasting | I |
| First visit after hospital discharge | Time between discharge and the first hospital visit, place of the first hospital visit, days of life during the first hospital visit | PC |
| Child-related factors | | |
| Perinatal conditions | Sex, twinning, birth weight, gestational age at birth, Apgar at 5 minutes, perinatal morbidity at birth, surgical morbidity at birth, syndrome | I |
|  | Readmission | PC, M |
| Feeding during NICU admission | Gastric tube feeding, use of parenteral nutrition, translactation (supplemental feeding tube device), type of diet | I |
| Breastfeeding-related factors | | |
| Mother | Intention to breastfeed during pregnancy and after birth; previous breastfeeding, guidance on breastfeeding in prenatal care  Difficulties with breastfeeding, duration of exclusive breastfeeding | I  I, PC, M |
| Health services | Assistance and support in breastfeeding in the face of difficulties, place of mother’s support | I, PC, M |
| Factors related to the introduction of artificial liquids | Age of introduction of water, tea or juice and reason for supply | PC, M |
| Factors related to the use of bottle and pacifier | use of a bottle, use of a pacifier, reason for the provision of the bottle and pacifier | I, PC, M |

**Captions:**

**I – During hospitalization after birth or transfer**

**PC – First visit after discharge**

**M – Monthly until the sixth month**
