# Supplementary material for: Breastfeeding patterns in cohort infants at a high-risk fetal, neonatal and child referral center in Brazil: a correspondence analysis
Source: BMC Pediatr. 2020 Aug 7;20:372. doi: 10.1186/s12887-020-02272-w (PMC7412808; doi:10.1186/s12887-020-02272-w)
Supplement: Supplementary file 2 — Additional file 2. Comparison between 928 participants and 75 non-respondents. Rio de Janeiro, Brazil, 2018. [file 12887_2020_2272_MOESM2_ESM.docx]

| **Additional file 2. Comparison between 928 participants and 75 non-respondents. Rio de Janeiro, Brazil, 2018.** | | | | | | | | | | |
| --- | --- | --- | --- | --- | --- | --- | --- | --- | --- | --- |
| **Characteristics** | **Participants** | | | | **Non-respondents** | | | | | **P-value** |
|  | **n** | **%** | **95% CI** | | | **n** | **%** | **95% CI** | |  |
| **Sex** |  |  |  |  | |  |  |  |  | 0.740 |
| Female | 445 | 48.0 | 44.6-51.2 | | | 38 | 50.7 | 38.8-62.4 | |  |
| Male | 483 | 52.0 | 48.7-55.3 | | | 37 | 49.3 | 37.5-61.1 | |  |
| **Twinning** |  |  |  |  | |  |  |  |  | 0.010 |
| No | 782 | 84.3 | 81.7-86.5 | | | 72 | 96.0 | 88.7-99.1 | |  |
| Twins / triplets / quadruplets | 146 | 15.7 | 13.4-18.2 | | | 3 | 4.0 | 0.8-11.2 | |  |
| **Gestational age** |  |  |  |  | |  |  |  |  | 1.000 |
| Higher or equal to 37 weeks | 719 | 77.5 | 74.6-80.1 | | | 58 | 77.3 | 66.2-86.2 | |  |
| Less than 37 weeks | 209 | 22.5 | 19.8-25.3 | | | 17 | 22.7 | 13.7-33.7 | |  |
| **Presence of perinatal morbidity** |  |  |  | | |  |  |  | | 0.895 |
| No | 546 | 58.8 | 55.5-62.0 | | | 43 | 57.3 | 45.3-68.6 | |  |
| Yes | 382 | 41.2 | 37.9-44.4 | | | 32 | 42.7 | 31.3-54.6 | |  |
| **Birth weight** |  |  |  |  | |  |  |  |  | 0.526 |
| Higher than 2.500 grams | 742 | 80.0 | 77.2-82.4 | | | 64 | 85.3 | 75.2-92.4 | |  |
| Between 1.500 and 2.500g | 149 | 16.1 | 13.7-18.5 | | | 9 | 12.0 | 5.6-21.5 | |  |
| Less than 1.500 grams | 37 | 4.0 | 2.8-5.4 | | | 2 | 2.7 | 19.3-40.9 | |  |
| **Delivery type** |  |  |  |  | |  |  |  |  | 0.039 |
| Transpelvian | 375 | 40.4 | 37.2-43.6 | | | 40 | 53.3 | 41.4-64.9 | |  |
| Cesarean | 553 | 59.6 | 56.3-62.7 | | | 35 | 46.7 | 35.0-58.5 | |  |
| **Schooling** |  |  |  |  | |  |  |  |  | 0.001 |
| Illiterate / incomplete elementary school | 95 | 10.3 | 8.3-12.3 | | | 17 | 23.0 | 13.7-33.7 | |  |
| Complete elementary school / incomplete  secondary school | 245 | 26.5 | 23.5-29.3 | | | 26 | 35.1 | 24.0-46.5 | |  |
| Complete secondary school/ incomplete  higher education | 496 | 53.6 | 50.1-56.6 | | | 27 | 36.5 | 25.2-47.9 | |  |
| Complete higher education | 90 | 9.7 | 7.8-11.7 | | | 4 | 5.4 | 1.4-13.0 | |  |
| **Parity** |  |  |  |  | |  |  |  |  | 0.482 |
| Primiparous | 453 | 49.1 | 45.5-52.0 | | | 40 | 54.1 | 41.4-64.9 | |  |
| Multiparous | 470 | 50.9 | 47.3-53.9 | | | 34 | 45.9 | 33.7-57.2 | |  |
| **Tobacco use during pregnancy** | 71 | 7.7 | 6.0-9.5 | | | 11 | 14.9 | 7.5-24.7 | | 0.053 |
| **Household income** |  |  |  |  | |  |  |  |  | 0.080 |
| Higher than two minimum wages | 473 | 61.3 | 47.6-54.2 | | | 25 | 48.1 | 22.8-45.1 | |  |
| Less than two minimum wages | 298 | 38.7 | 29.1-35.2 | | | 27 | 51.9 | 25.2-47.9 | |  |
| **Working outside the home** | 422 | 45.8 | 42.2-48.7 | | | 27 | 37.0 | 25.2-47.9 | | 0.181 |
| **Place of hospitalization** |  |  |  |  | |  |  |  |  | 0.111 |
| Rooming-in | 628 | 67.7 | 64.5-70.6 | | | 46 | 61.3 | 49.3-72.3 | |  |
| Neosurgical ICU | 47 | 5.1 | 3.7-6.6 | | | 8 | 10.7 | 4.7-19.9 | |  |
| Neonatal ICU | 252 | 27.2 | 24.3-30.1 | | | 21 | 28.0 | 18.2-39.5 | |  |
| **Exclusive breastfeeding at discharge** | 593 | 65.3 | 60.7-66.9 | | | 46 | 62.2 | 49.3-72.3 | | 0.464 |

P-value < 5%. ICU = Intensive Care Unit. IC = interval confidence
